# The diversity of myeloid immune cells shaping wound repair and fibrosis in the lung

**DOI:** 10.1002/reg2.97

**Published:** 2018-02-23

**Authors:** Laura Florez‐Sampedro, Shanshan Song, Barbro N. Melgert

**Affiliations:** ^1^ Department of Pharmacokinetics, Toxicology and Targeting Groningen Research Institute for Pharmacy, University of Groningen Antonius Deusinglaan 1 9713 AV Groningen The Netherlands; ^2^ Department of Chemical and Pharmaceutical Biology Groningen Research Institute for Pharmacy University of Groningen Antonius Deusinglaan 1 9713 AV Groningen The Netherlands; ^3^ University Medical Center Groningen, Groningen Research Institute for Asthma and COPD University of Groningen Hanzeplein 1 9713 GZ Groningen The Netherlands

**Keywords:** dendritic cells, fibrocytes, macrophages, monocytes, neutrophils

## Abstract

In healthy circumstances the immune system coordinates tissue repair responses in a tight balance that entails efficient inflammation for removal of potential threats, proper wound closure, and regeneration to regain tissue function. Pathological conditions, continuous exposure to noxious agents, and even ageing can dysregulate immune responses after injury. This dysregulation can lead to a chronic repair mechanism known as fibrosis. Alterations in wound healing can occur in many organs, but our focus lies with the lung as it requires highly regulated immune and repair responses with its continuous exposure to airborne threats. Dysregulated repair responses can lead to pulmonary fibrosis but the exact reason for its development is often not known. Here, we review the diversity of innate immune cells of myeloid origin that are involved in tissue repair and we illustrate how these cell types can contribute to the development of pulmonary fibrosis. Moreover, we briefly discuss the effect of age on innate immune responses and therefore on wound healing and we conclude with the implications of current knowledge on the avenues for future research.

## 1 INTRODUCTION

**Figure 1:**
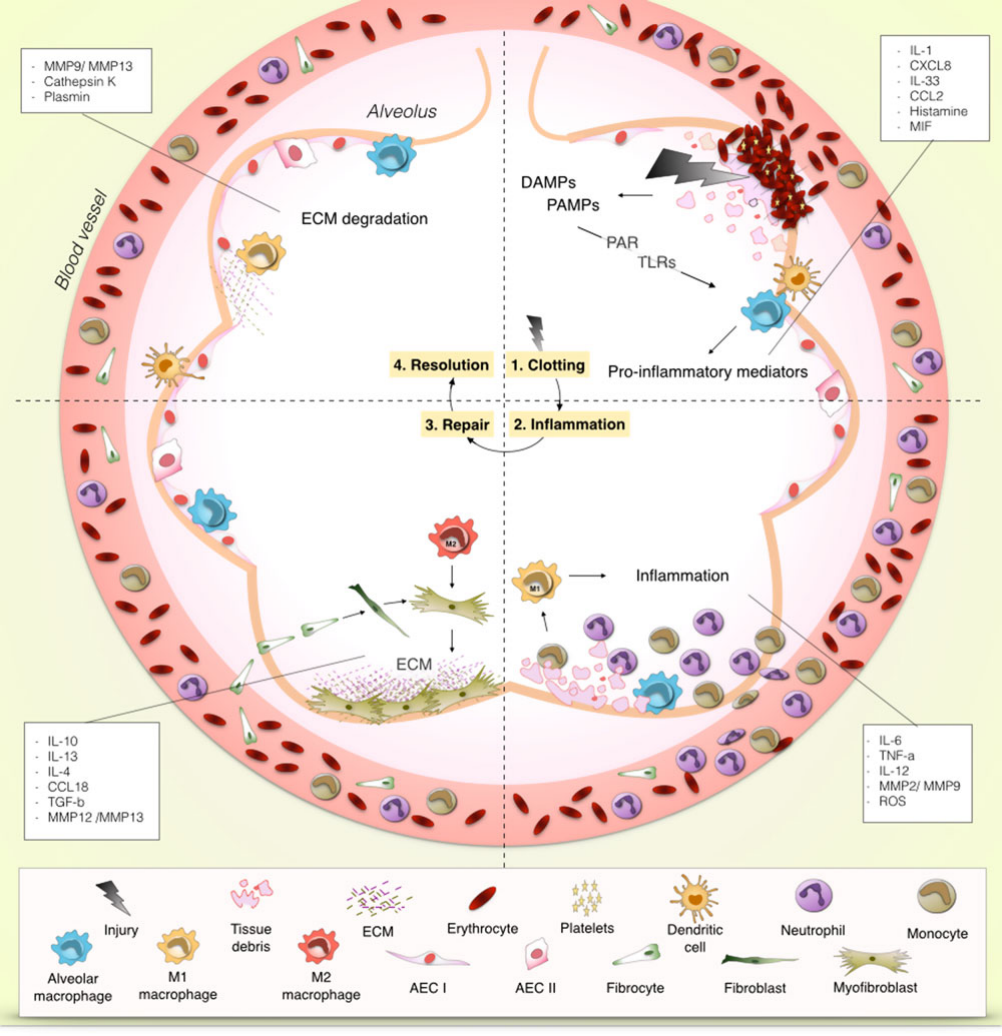
**Phases of tissue repair in the context of myeloid cells**. (1) Clotting (top right) is the first step after injury takes place. Damage to alveolar epithelial cells (AEC) leads to the aggregation of erythrocytes and platelets that form a blood clot to contain spreading of the damage. The destruction of the tissue causes release of damage‐associated molecular patterns (DAMPs), or allows the entrance of microorganisms and thereby pathogen‐associated molecular patterns (PAMPs). Local cells such as dendritic cells, macrophages, and AECs are activated by DAMPs and PAMPs through toll‐like receptors (TLRs) and protease‐activated receptors (PARs) and produce the first round of pro‐inflammatory mediators. (2) Inflammation (bottom right) involves the infiltration of neutrophils and monocytes that respond to the pro‐inflammatory stimuli that were produced by resident lung cells. Phagocytes such as neutrophils and macrophages remove tissue debris and potentially threatening particles. In this pro‐inflammatory environment, monocytes and resident macrophages can differentiate into M1 macrophages that further promote inflammatory responses by the production of pro‐inflammatory mediators such as IL‐6 and TNF‐α. (3) Repair (bottom left) is the phase in which inflammatory responses subside and turn into repair responses through the effects of anti‐inflammatory and pro‐repair cytokines such as IL‐10 and TGF‐β. In this stage fibrocytes enter the tissue and differentiate into fibroblasts that proliferate and turn into the more contractile myofibroblasts. Myofibroblasts produce extracellular matrix (ECM) to close the open wound and form a scar. M2 macrophages predominate in this stage and produce mediators that contribute to the proliferation of fibroblasts and the deposition of ECM. (4) Resolution (top left) refers to the last phase in tissue repair in which excess ECM is degraded to make space for new cells. At the end of this stage the tissue has regained its structure and function

Tissue repair is an essential process that allows the replacement of damaged cells and the restoration of organ function, which is key for survival (Wynn, 2007). When a tissue is harmed, the immune system acts to contain potential threats and to re‐establish function and structure of the affected organ (Li, Chen, & Kirsner, 2007). Tissue damage can be caused by microbial infections, the exposure to toxic compounds, burns, and mechanical trauma, among other factors. Although the details of the immune events might vary depending on the nature of the injury, in general the phases leading to tissue recovery are rather conserved. These phases are clotting, inflammation, tissue repair, and resolution and return to tissue homeostasis (see Fig. 1) (Li et al., 2007). The daily exposure to airborne microorganisms and particles makes the lung a susceptible target for tissue injury, which is why evolution provided the mammalian lung with an elaborate immune system (Nicod, 2005). The optimal function of the lung relies on a regulated balance between immune responses, tissue repair, and tissue function, accommodating protection from infection and injury without considerably affecting tissue structure and function. This makes the lung an interesting immune center and therefore here we focus on the lung as the model organ to exemplify the general steps associated with typical tissue injuries and the alterations of innate immune responses in wound healing and fibrosis development. Whenever available we include data from clinical studies involving patients with pulmonary fibrosis of either unknown origin (idiopathic pulmonary fibrosis or IPF) or of known origin and from well‐known mouse models of pulmonary fibrosis.

## 2 TISSUE REPAIR AND FIBROSIS DEVELOPMENT

### 2.1 Phases of tissue repair

The events associated with acute injury involve the activity of the innate part of the immune system (Fig. 1). Infiltration of pathological microorganisms or destruction of tissue results in expression of molecular patterns that alarm the immune system about danger. In the case of an infection these molecules are known as pathogen‐associated molecular patterns (PAMPs), and in the case of destruction of tissue they are known as damage‐associated molecular patterns (DAMPs) (Davidovich, Kearney, & Martin, 2014; Medzhitov, 2007). DAMPs are usually molecules that are found in the intracellular milieu under normal conditions and thus, once found in the extracellular space, are a sign of tissue damage (e.g., heme and thrombin) (Chen & Nuñez, 2010). The release of DAMPs and PAMPS will activate resident cells such as macrophages, dendritic cells (DCs), mast cells, epithelial cells, natural killer cells and innate lymphoid cells through their sensors of these molecules, i.e., toll‐like receptors (TLRs). Their activation will result in the production of other soluble mediators, such as histamine, interleukins (IL, e.g. IL‐1 and IL‐33), and other cytokines, and chemokines (e.g., macrophage migration inhibitory factor [MIF], CCL2, and CXCL8, which function as an alarm to activate and recruit other cells of the immune system to the affected area (Hussell & Bell, 2014; Julier, Park, Briquez, & Martino, 2017; Kurowska‐Stolarska et al., 2009; Zimmermann, Trautwein, & Tacke, 2012).

The release of DAMPs and PAMPs occurs in parallel with activation of the coagulation cascade that is initiated by damage to blood vessels. This is the beginning of the clotting phase. Clotting prevents blood loss and protects the open wound from further exposure. Clotting also has a direct effect on resident tissue macrophages and leads to their activation through exposure to free heme from coagulation and through thrombin‐induced cleavage of protease‐activated receptors found on macrophages and other resident cells (Asokananthan et al., 2002; Colognato et al., 2003; Dutra & Bozza, 2014; Mercer & Chambers, 2013; Minutti, Knipper, Allen, & Zaiss, 2016). The second phase, known as inflammation, begins with infiltration of recruited leukocytes to the wound. These leukocytes are mostly neutrophils and monocytes that phagocytose microorganisms, dying cells and cell debris, thus preventing the spread of damage and pathogens (Dale, Boxer, & Conrad Liles, 2008). Some of these monocytes differentiate into macrophages that initially also promote inflammation. Eventually the inflammation phase declines and progresses towards a repair phase, in which anti‐inflammatory and pro‐repair cytokines such as IL‐10 and transforming growth factor beta (TGF‐β) activate myofibroblasts. This leads to the deposition of extracellular matrix (ECM) for formation of scar tissue that serves as a temporary protection and scaffold for newly formed tissue (Boorsma, Draijer, & Melgert, 2013). At this point parenchymal cells start regrowing, which sets the beginning of the last phase known as resolution and return to homeostasis. During this phase, excess ECM is removed by macrophages and fibroblasts through the production of proteases such as cathepsins, matrix metalloproteinases (MMPs), and plasmin, and parenchymal cell growth is stimulated for the re‐establishment of tissue structure and function (Adhyatmika, Putri, Beljaars, & Melgert, 2015; Boorsma et al., 2013). The duration of each of these phases is tissue‐dependent and may be further influenced by external conditions and individual factors (Guo & Dipietro, 2010).

Wound healing in adult mammals can only restore structure and function to some degree as we have a limited regenerative capacity. Only our liver and skeletal muscles can recover from significant tissue damage (Corona et al., 2013; Michalopoulos, 2013; Taub, 2004). Mammalian embryos, on the other hand, are capable of scar‐free repair but they lose this ability soon after birth (Ferguson & O'Kane, 2004; Kishi, Okabe, Shimizu, & Kubota, 2012; Porrello et al., 2011). It has been suggested that the complexity and specialization of the immune system in animals is inversely correlated to the ability for tissue regeneration, as smaller animals like amphibians can regenerate completely severed limbs (Godwin & Rosenthal, 2014; Godwin, Pinto, & Rosenthal, 2016; Julier et al., 2017). Studies in frogs suggest that the adaptive immune system may be involved in the limitation of tissue regeneration (Mescher et al., 2007). Other amphibians, young frogs, and newborn humans either lack or have immature adaptive immunity and have greater regenerative potential (Godwin & Rosenthal, 2014; Mescher et al., 2007). This suggests that the key to scar‐free healing and active regeneration in adult mammals relies on the innate immune system.

### 2.2 Dysregulated wound healing in the pathogenesis of fibrosis

Despite the limited capacity for regeneration, healthy individuals are capable of healing in most situations and eventually tissue homeostasis is restored. However, dysregulation of any of the cells or stages of wound healing or continuous repetitive injury could lead to pathological outcomes such as cancer, tissue destruction, and/or fibrosis. Interestingly, repetitive exposure to the same harmful agent does not always lead to the same outcome in all individuals. Such is the case for cigarette smoke exposure, which does not always lead to a pathological condition but it has been associated with the development of lung cancer, chronic obstructive pulmonary disease (COPD), and pulmonary fibrosis (Jindal et al., 2006; Lubin et al., 1984; Madan, Matalon, & Vivero, 2015). This variability in wound healing outcomes is partly due to the genetic and epigenetic background of each individual and other intrinsic factors such as age, gender, comorbidities, and many other factors (reviewed by Guo & Dipietro, 2010). Age in particular has a great influence on the healing response, as many components of the immune system change over time and the response to insults tends to be altered (reviewed by Boe, Boule, & Kovacs, 2016). This is partly the reason why some diseases such as emphysema and pulmonary fibrosis mostly develop at older age.

Pulmonary fibrosis can develop as an end stage disease in many other types of disorders, such as scleroderma, viral infections, disease caused by exposure to harmful substances or drugs (such as silicosis induced by exposure to silica), but often its cause is unknown (IPF) (Wynn, 2011). The fibrosis is caused by a dysregulation in wound healing responses and the subsequent accumulation of ECM, followed by changes in lung structure that lead to organ malfunction and ultimately lethal respiratory failure (Camelo, Dunmore, Sleeman, & Clarke, 2014; Spagnolo, Maher, & Richeldi, 2015; Wynn, 2011).

Once fibrosis develops, there are few available treatment options. In recent clinical trials, pirfenidone, nintedanib, and *N*‐acetylcysteine have been shown to be effective in reducing the functional decline and disease progression in pulmonary fibrosis (Staitieh, Renzoni, & Veeraraghavan, 2015). However, neither pirfenidone nor nintedanib nor *N*‐acetylcysteine is a cure for the disease and most patients continue to progress despite treatment. Therefore the average survival time after diagnosis is only 3−5 years (Spagnolo et al., 2015).

**Figure 2:**
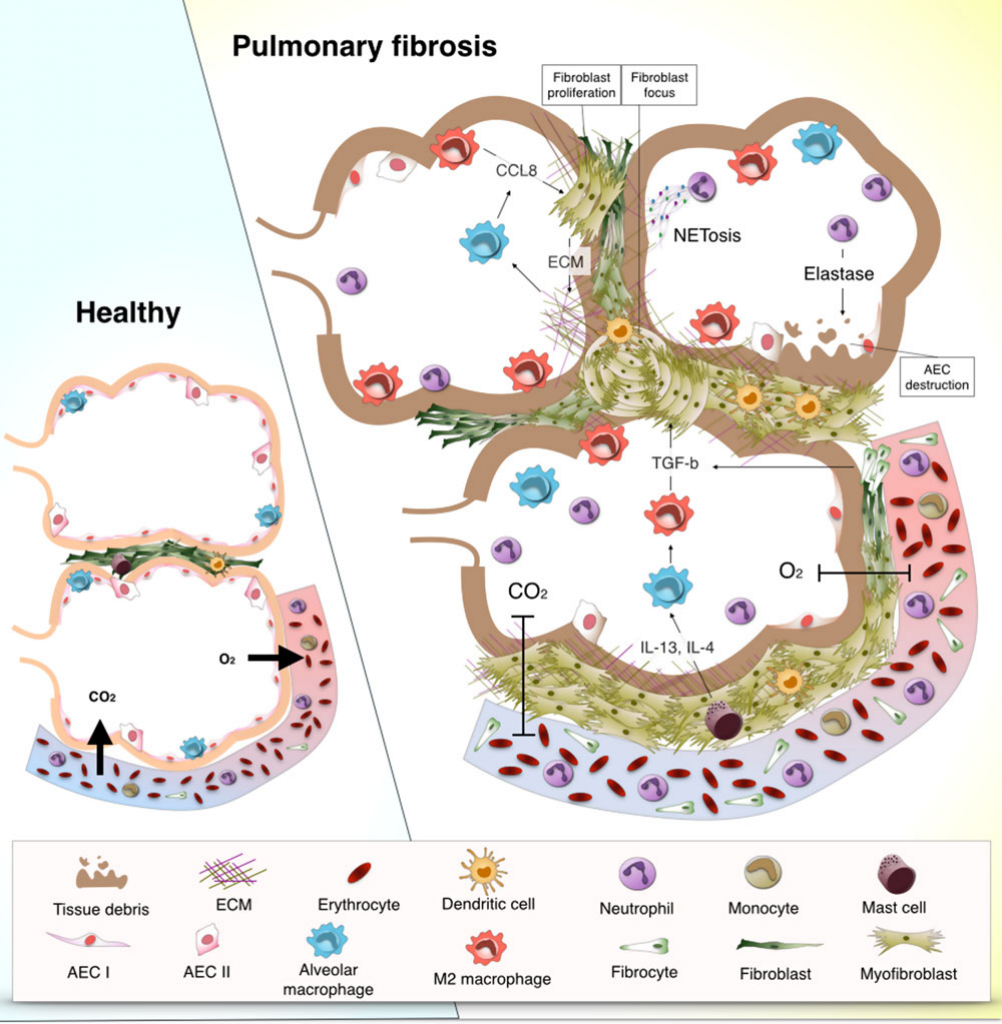
**Pathophysiology of pulmonary fibrosis in the context of myeloid cells**. The pathogenesis of pulmonary fibrosis is characterized by an exaggerated repair response to lung injury. These alterations in repair responses include proliferation of fibroblasts in the area of the injury and differentiation towards myofibroblasts that can form fibroblast foci. Dendritic cells accumulate in fibroblast foci in an immature form due to the influence of fibroblasts, which decreases T cell activation and proliferation. The predominant Th2 cytokine profile (e.g., IL‐4, IL‐13), produced by mast cells among others, promotes the polarization of macrophages towards an M2 phenotype. In turn, M2 macrophages produce soluble mediators such as TGF‐β and CCL8 that lead to fibroblast proliferation and to their differentiation into myofibroblasts and subsequent production of extracellular matrix (ECM). Moreover, deposition of ECM stimulates CCL8 production by alveolar macrophages resulting in a profibrotic vicious cycle. High numbers of neutrophils in fibrotic lung tissue contribute to the fibrotic process by perpetuating tissue damage and epithelial destruction via the production of elastase and possibly also by NETosis. High percentages of circulating fibrocytes are characteristic of pulmonary fibrosis. These circulating fibrocytes enter lung tissue and develop into fibroblasts that in the fibrotic environment differentiate into myofibroblasts. Additionally, fibrocytes contribute to the fibrotic process by producing TGF‐β and other mediators that contribute to the redundant and uncontrolled pro‐repair environment of the fibrotic lung

The pathogenesis of pulmonary fibrosis is usually characterized by injury to alveolar epithelial cells, recruitment and activation of (myo)fibroblasts, and subsequent production of ECM, and the dysregulated activity of macrophages (Boorsma et al., 2013; Kolahian, Fernandez, Eickelberg, & Hartl, 2016; Spagnolo et al., 2015; Wynn, 2011). Fibroblasts hyperproliferate in the fibrotic lung forming what is known as fibrotic foci, which are thought to represent points of injury (Knudsen, Ruppert, & Ochs, 2017). In fibrosis, fibroblasts differentiate into more contractile and apoptosis‐resistant myofibroblasts promoting the fibrotic process. These two types of cells produce immature forms of collagen and other ECM proteins that promote a pro‐repair macrophage polarization (M2), which in turn produces CCL18 that induces fibroblast activation and more ECM production (Kolahian et al., 2016; Prasse et al., 2006). This is one of the many examples of cell interactions that create a profibrotic vicious circle that is probably promoting fibrosis progression. A summary of the processes playing a role in the pathogenesis of pulmonary fibrosis is depicted in Figure 2.

Although cigarette smoke exposure can contribute to pulmonary fibrosis, the nature of the initial injury is not always known, particularly in IPF (Madan et al., 2015). Moreover, it is also unclear why the lung is unable to restore tissue homeostasis and instead continues the repair process to the point of turning into fibrosis. It is possible, however, that epigenetic regulations following repetitive injury may be the cause of the change from repair to fibrosis. Most types of fibrosis do not present with an inflammatory component, but it is hypothesized that fibrosis is initiated with a lung injury and a subsequent inflammatory response, which will eventually be altered and skewed towards an extreme pro‐repair response (Wynn, 2011). This may explain why anti‐inflammatory and immunosuppressive agents were shown to be ineffective for patients with pulmonary fibrosis, because at the moment of diagnosis they are usually beyond inflammation and suffering from severe fibrotic disease (Staitieh et al., 2015). As mentioned before, due to the impressive scar‐free tissue repair of small amphibians lacking adaptive immunity, we believe that the key to proper healing of fibrosis relies on the innate immune system, i.e., cells of myeloid origin. Therefore, understanding how these myeloid cells act during functional wound healing versus fibrotic healing can provide an insight into future therapeutic measures.

## 3 MYELOID CELLS IN TISSUE REPAIR AND FIBROSIS IN THE LUNG

The term “myeloid,” from the ancient Greek *Muelos‐* (marrow) and *‐eides* (likeness), refers to cells that resemble those in bone marrow. In a hematopoietic context, the myeloid line actually refers to those cells that in fact originate from bone marrow progenitors and form the granulocytic and monocytic lineages, but not the lymphoid lineage (Kawamoto & Minato, 2004). Myeloid progenitors can develop into innate immune cells that compose the primary response against microorganisms and injury. Therefore, myeloid cells have been the focus of interest in studies of wound healing and fibrosis development (Doulatov, Notta, Laurenti, & Dick, 2012; Kawamoto & Minato, 2004). Myeloid cells can be further classified by the presence or absence of granules, observed after leukocyte staining. They are divided into granulocytes, i.e., neutrophils, mast cells, eosinophils, and basophils, and agranulocytes or monocytic cells, i.e., monocytes and their derived cells, macrophages, DCs, and fibrocytes (Kay, 2016). We shall use this classification to present these cell types and their involvement in wound healing and fibrosis.

### 3.1 Monocytes and monocyte‐derived cells

#### 3.1.1 Monocytes

Monocytes are mononuclear leukocytes derived from common monocyte progenitor cells in bone marrow after stimulation with macrophage colony stimulating factor (M‐CSF) (Auffray, Sieweke, & Geissmann, 2009; Hettinger et al., 2013). Once in blood, monocytes account for 10% and 4% of leukocytes in human and mouse, respectively (Das et al., 2015).

During tissue injury, monocytes are commonly known to arrive at the area of injury right after neutrophils. Older studies estimated that monocytes arrive 1−3 days after neutrophils, but more recent investigations have found that monocytes can arrive at an injured tissue within the first hours after tissue damage simultaneously with neutrophil infiltration (Dal‐Secco et al., 2015; Gurtner, Werner, Barrandon, & Longaker, 2008; Rodero et al., 2014). To be noted, these results originate from studies using mouse models of sterile injuries of the skin and the liver and it is not known yet whether this is also happening in other tissues or in all types of injury (Dal‐Secco et al., 2015; Rodero et al., 2014). The role of monocytes during wound healing is to some extent similar to that of neutrophils since they also phagocytose tissue debris and pathogens (similarities and differences between these cell types have been reviewed elsewhere (Dale et al., 2008). However, monocytes function in a more complex way than neutrophils, as they also give origin to other cells important for wound healing, such as macrophages, DCs, and fibrocytes.

During monocyte maturation in mice, two monocyte subpopulations are found. Monocytes formed in bone marrow are characterized by high expression of Ly6C (Ly6C^hi^). These Ly6C^hi^ monocytes can migrate from bone marrow to blood after stimulation with CCL2 and CCL7, which is why they have high expression of the chemokine receptor CCR2 (Auffray et al., 2009; Tsou et al., 2007). Ly6C^hi^ monocytes, also known as classical monocytes, have a pro‐inflammatory phenotype and thus are the ones usually found during acute injury. Classical monocytes can develop into nonclassical monocytes, which are characterized as Ly6C^low^ and CX3CR1‐positive with a prohealing phenotype (Hanna et al., 2015; Hettinger et al., 2013; Yona et al., 2013). In steady state conditions classical monocytes are found patrolling extravascular tissues while nonclassical monocytes are found patrolling blood vessels (Auffray et al., 2007; Carlin et al., 2013). This patrolling behavior, at least in the case of the nonclassical monocytes, was shown to provide immune surveillance to the surrounding tissues since they were shown to extravasate rapidly into a tissue that has been submitted to sterile, toxic, or infectious injury (Auffray et al., 2007). Interestingly, these nonclassical patrolling monocytes were found enriched in the lung microvasculature and were shown to have a protective effect against cancer by reducing tumor metastasis through the recruitment of other immune cells (Hanna et al., 2015).

In humans, monocytes possess a different molecular nomenclature but it seems from the limited studies available in humans that they behave similarly to those in mice. Human monocytes are classified based on their expression of CD14 (a membrane receptor for lipopolysaccharide [LPS]) and CD16 (a low affinity immunoglobulin G receptor). With these two markers it is possible to identify three monocyte populations in human blood: CD14++CD16− (classical monocytes), CD14+CD16+ (intermediate monocytes), and CD14+CD16++ (nonclassical monocytes), accounting for around 85%, 5% and 9% of all monocytes, respectively (Glezeva, Horgan, & Baugh, 2015; Hulsmans, Sam, & Nahrendorf, 2016; Shi & Pamer, 2011; Wang & Xia, 2013; Woollard & Geissmann, 2010; Ziegler‐Heitbrock & Hofer, 2013). Additionally, just as in mice the human classical subtype is CCR2+ and the nonclassical subtype is CX3CR1+ (Tacke & Randolph, 2006). Similar to what occurs in mice, human nonclassical CD14+CD16− monocytes are thought to originate from classical CD14++CD16− monocytes in blood in an M‐CSF‐dependent way (Korkosz, Bukowska‐strakova, Sadis, Grodzicki, & Siedlar, 2012; Weiner et al., 1994; West et al., 2012). This differentiation happens via the formation of the intermediate phenotype, which has been associated with different pathological conditions such as cardiovascular disease, trauma, sepsis, and autoimmunity (Rossol, Kraus, Pierer, Baerwald, & Wagner, 2012; Tapp, Shantsila, Wrigley, Pamukcu, & Lip, 2012; Urra et al., 2009; West et al., 2012). It is hypothesized that increased numbers of intermediate monocytes in these diseases are triggered by tissue damage and could be functionally associated with tissue repair and regeneration (Schauer et al., 2014).

Monocytes are well known for being possible progenitors of macrophages and DCs. Due to their ability to change into these cells, monocytes can also indirectly contribute to disease. However, there is evidence that they can influence wound‐healing processes while maintaining their monocytic phenotype (Dal‐Secco et al., 2015). Jakubzick et al. (2013) showed that monocytes are able to move through tissues and migrate towards lymph nodes maintaining their monocytic phenotype without differentiating to DCs or macrophages. Mouse studies suggest that monocytes infiltrate the injured tissue in two waves: the first wave involves an influx of classical Ly6C^hi^ CCR2+ monocytes contributing to inflammation and angiogenesis followed by a later influx of nonclassical Ly6C^low^ CX3CR1+ monocytes that contribute to scar formation (Ishida, Gao, & Murphy, 2008; Willenborg et al., 2012). In the context of liver injury in particular it was shown by Dal‐Secco and colleagues that monocytes themselves can restore homeostasis after tissue injury without differentiating into macrophages (Dal‐Secco et al., 2015). These authors showed via intravital microscopy that classical Ly6C^hi^ CCR2+ monocytes that infiltrated mouse liver tissue after a sterile injury changed their phenotype in situ towards nonclassical Ly6C^low^ CX3CR1+ monocytes. This change was IL‐4 and IL‐10 dependent and associated with a decrease in dead cells and tissue debris. This suggests that monocytes are capable of re‐establishing homeostasis of tissues without relying on differentiation to macrophages or the recruitment of other cells. Furthermore, this could mean that what initially was conceived as a process involving two waves of infiltration might actually be a transformation of inflammatory classical monocytes towards a nonclassical prohealing phenotype. It is possible, however, that the resolution of injury observed in this study also had a contribution from local macrophages. It is not known whether this classical‐to‐nonclassical monocyte differentiation in situ occurs in the lung and other tissues and with other types of injury. These observations challenge the idea of some researchers to refer to infiltrating tissue monocytes as macrophages and reflect the possible influence of monocytes on the fate of a tissue after injury. To be noted, although these observations originate from mouse studies, it is likely that this in situ monocyte conversion also occurs in humans due to the similarities in the classical and nonclassical subtypes of monocytes in mouse and humans.

In the context of fibrosis there is some evidence that classical Ly6C^hi^ monocytes promote the fibrotic process. In a mouse model of pulmonary fibrosis, the depletion of classical Ly6C^hi^ monocytes resulted in less fibrosis, and additionally the adoptive transfer of these monocytes aggravated fibrosis (Gibbons et al., 2011). Although depletion of classical Ly6C^hi^ monocytes led to a decrease in the numbers of profibrotic macrophages (Ym1‐positive alternatively activated macrophages), this study was not able to prove that these macrophages originate from classical Ly6C^hi^ monocytes. Another study using two mouse models of liver fibrosis showed that inhibiting the accumulation of classical Ly6C^hi^ monocytes during the resolution phase of fibrosis accelerated scar resolution (Baeck et al., 2014). This shows that also in liver fibrosis the classical Ly6C^hi^ monocytes contribute to the fibrotic process. Gibbons and colleagues hypothesized that classical Ly6C^hi^ monocytes could contribute to the fibrotic process by means of cell‐to‐cell interactions with macrophages and possibly even myofibroblasts in the fibrotic foci of the developing fibrotic lung. This is supported by Mewhort and colleagues, who showed that human monocytes from peripheral blood (which were 95% classical monocytes) induce the activation of cardiac fibroblasts towards myofibroblasts via direct cell‐to‐cell interaction, to a greater extent than the effect of TGF‐β on fibroblasts (Mewhort et al., 2016).

Most studies on immune responses in fibrosis have focused on the influence of monocyte‐derived macrophages and not on monocytes per se, and it is therefore still unclear what the actual contributions of the classical Ly6C^hi^ and nonclassical Ly6C^lo^ monocytes are to the fibrotic process. Considering that monocytes are one of the first cell types to arrive at injured tissue, and based on the different abilities they have, they play an important role in defining whether an initial insult is suitably repaired or develops into a chronic condition. It is possible that Ly6C^hi^ profibrotic monocytes are the origin of profibrotic macrophages. Perhaps modern techniques like intravital microscopy and the use of fluorescent monocytes (labeled or genetically fluorescent) can clarify this.

#### 3.1.2 Macrophages

Macrophages are tissue‐resident myeloid leukocytes belonging to the innate immune system that are key in maintaining tissue homeostasis. They have heterogeneous functions ranging from phagocytosis of microorganisms, ECM components, or dying cells to the production of cytokines for the modulation of other cells involved in immune responses (Murray & Wynn, 2011). Overall, they are capable of maintaining tissue homeostasis by developing into different phenotypic populations according to what the tissue environment requires at that moment. Mouse studies depleting macrophages during different stages after tissue injury have shown that macrophages are important for the inflammatory, the prohealing, as well as the resolution stages of wound healing (Duffield et al., 2005; Gibbons et al., 2011). Studies with salamanders have shown that their great regenerative capacity is actually dependent on macrophages (Godwin, Pinto, & Rosenthal, 2013). Godwin and colleagues showed in adult salamanders that the regeneration of whole limbs after amputation was hampered upon macrophage depletion, thus reflecting the importance of macrophages in tissue repair.

Depending on their phenotype and role, macrophages can be broadly classified as M1 and M2, although in reality there is a spectrum of phenotypes (Gautier et al., 2012; Hume, 2015). M1 macrophages, also known as classically activated macrophages, are pro‐inflammatory, associate with Th1 inflammation, and are activated immediately after tissue injury or infection. M2 macrophages, or alternatively activated macrophages, associate with Th2 inflammation, resolution of inflammation, and tissue repair (Martinez & Gordon, 2014; Mills, 2015). M2 macrophages can be generated by many stimuli, which is why this type is often subdivided into three classes: M2a, activated by IL‐4 or IL‐13; M2b, activated by immune complexes; M2c, activated by IL‐10, TGF‐β and glucocorticosteroids (Martinez & Gordon, 2014; Martinez, Sica, Mantovani, & Locati, 2008; Murray et al., 2014). In healthy conditions, M1 macrophages would be involved in the first stages of wound healing, due to their pro‐inflammatory phenotype, and M2 macrophages would follow leading to a decrease in inflammation and the beginning of a repair process that eventually will re‐establish homeostasis and tissue structure (Adhyatmika et al., 2015; Braga, Agudelo, & Camara, 2015). Additionally, there are some indications that M1 macrophages may also be important during resolution of fibrosis. Our studies on the liver in the context of liver fibrosis have shown that M1‐polarized macrophages persist during resolution of liver fibrosis, while the number of M2 macrophages decreases (Beljaars et al., 2014). In addition, forcing liver macrophages to polarize towards M2 by macrophage‐specific delivery of corticosteroids aggravates the fibrotic process in the liver (Melgert et al., 2001). Gibbons et al. (2011) and He, Ryan, Murthy, and Carter (2013) have shown similar findings for the lung.

Macrophages can originate from blood monocytes during inflammation after monocytes have infiltrated the injured tissue (Murray & Wynn, 2011). However, not all tissue macrophages originate from monocytes. It is now known that many tissue‐resident macrophages, including alveolar macrophages, originate from yolk sac macrophages or from erythro‐myeloid progenitors in the fetal liver (Ginhoux & Guilliams, 2016; Ginhoux et al., 2010; Guilliams et al., 2013; Hoeffel et al., 2012, 2015; McGrath et al., 2015; Schulz et al., 2012). These tissue‐resident macrophages in steady state conditions are self‐renewing and long‐lived, but under inflammatory conditions they can be supplemented by infiltrating monocytes (Epelman et al., 2014; Heidt et al., 2014; Hilgendorf et al., 2014; Movita et al., 2015). It is important to make a distinction between these two types of macrophages, as they may function in different and even opposite ways in some conditions (Gundra et al., 2014; Vannella et al., 2014; Zaslona et al., 2014).

In pathological conditions or after repeated tissue insults the balance, interactions, and roles of the different macrophage subsets may be altered. Overall, it is known that many lung diseases present with altered numbers and functions of macrophages (reviewed by Boorsma et al., 2013). The particular case of pulmonary fibrosis is thought to associate with higher numbers of M2 macrophages, reflected by the high expression of M2 markers in IPF lavage fluid and in mouse models for pulmonary fibrosis (Boorsma et al., 2013; Gibbons et al., 2011; Pechkovsky et al., 2010), suggesting an attempt to repair the tissue. This is not surprising as M2 macrophages associate with repair processes and may directly stimulate myofibroblasts via TGF‐β to produce and deposit the ECM that characterizes the fibrotic process (Conway & Hughes, 2012). An uncontrolled increase in this activity could turn a “prohealing” activity into a “profibrotic” effect. This notion is also supported by the study from Gibbons and colleagues, in which they demonstrate that alternatively activated M2 macrophages are directly involved in the development of pulmonary fibrosis (Gibbons et al., 2011). Considering these data, it is understandable why glucocorticosteroid treatment can worsen pulmonary fibrosis, as this treatment activates a class of M2 macrophages. However, it is unknown whether the origin of these profibrotic macrophages in pulmonary fibrosis is from resident alveolar or interstitial macrophages or from infiltrating monocytes. It has been suggested that, in liver fibrosis, both resident liver macrophages (Kupffer cells) and monocyte‐derived macrophages contribute to the development of fibrosis (Baeck et al., 2014; Pradere et al., 2013). It still needs to be elucidated whether it is the same for pulmonary fibrosis. Interestingly, it has been shown in a mouse model of asthma that alveolar macrophages control inflammation, while monocyte‐derived macrophages promote inflammation after exposure to house dust mite (Zaslona et al., 2014). Although this is not reflecting what occurs in pulmonary fibrosis, it suggests that upon exposure to inflammatory triggers lung‐resident macrophages may play an opposite role to that of monocyte‐derived macrophages and this may also be relevant for pulmonary fibrosis.

To conclude, macrophages are multifaceted leukocytes that are key for wound healing. They can present differentially polarized phenotypes based on the triggers they are exposed to and the microenvironment of the niche they belong to. They are involved in the inflammatory, the healing, and the resolution phase of tissue repair, and they can exist as resident tissue macrophages originating during embryonic development or they could develop from infiltrating monocytes. Their different origins influence their behavior and possibly their involvement in pathological conditions. However, the exact contribution of each of these macrophages (i.e., tissue‐resident vs. recruited) in fibrosis, particularly in the lung, still needs to be clarified.

#### 3.1.3 Dendritic cells

DCs mature from myeloid progenitor cells after stimulation with granulocyte macrophage colony stimulating factor (GM‐CSF), IL‐4, tumor necrosis factor alpha (TNF‐α), and FMS‐like tyrosine kinase 3 ligand (FLT3L) (Ardavín, 2003; Ardavín et al., 2001). DCs are distributed through most lymphoid and nonlymphoid tissues, acting as sentinels, and are also present in peripheral blood, constituting 0.1−1% of all mononuclear cells (Orsini et al., 2012). Generally, lung resident DCs can be divided into two major classes: plasmacytoid DCs (pDCs) and conventional or classical DCs (cDCs). Under inflammatory conditions, monocyte‐derived DCs (moDCs) can also be found in the lung as well (Ganguly, Haak, Sisirak, & Reizis, 2013; Kim & Lee, 2014).

cDCs originate from hematopoietic stem cells in bone marrow that develop into lineage‐restricted macrophage‐DC progenitors, which in turn differentiate into common DC progenitors (Naik et al., 2007; Sathe et al., 2014). These common DC progenitors give rise to pre‐DCs that migrate into peripheral tissues and locally differentiate into mature cDCs mainly dependent on FLT3L (Kopf, Schneider, & Nobs, 2015). cDCs are dedicated antigen‐presenting cells with a high expression of major histocompatibility complex class II (MHCII).

pDCs also originate from hematopoietic stem cells but, in contrast to cDCs, pDC development occurs completely in bone marrow and mature pDCs migrate to peripheral tissues (Reizis, Bunin, Ghosh, Lewis, & Sisirak, 2011). M‐CSF and IL‐7 contribute to pDC development together with FLT3L (Waskow et al., 2008). pDCs express relatively low levels of MHCII and rapidly produce type I interferons (IFN) following activation through nucleic acid sensing TLRs (Ardavín, 2003; Ganguly et al., 2013).

During inflammation, classical Ly6C^hi^ and nonclassical Ly6C^lo^ monocytes can serve as precursors for moDCs and are recruited to the lung by pro‐inflammatory chemokines such as CCL2 and CCL7 and the cytokine CSF‐1 (Hoffmann et al., 2016). However, it is difficult to distinguish between moDCs and cDCs due to their similar surface markers. Overall, DCs are the primary professional antigen presenting cells and they connect the innate and adaptive immune system via uptake, transport, processing, and presentation of antigens to T cells, and cytokine production (Guilliams, Lambrecht, & Hammad, 2013).

DCs can take up and remove foreign particles and pathogens in a similar way to macrophages, which is partly why they also play an important role in wound healing. Resident DCs can also get activated via TLRs after sensing local danger signals produced by injury (Hartl et al., 2012). Additionally, pDCs can secrete type I IFNs to promote wound healing (Gregorio et al., 2010). The overall importance of DCs in wound healing is demonstrated in DC‐deficient mice that present with a significant delay in early wound healing after contact burns (Gregorio et al., 2010). Moreover, compared to control mice, DC‐deficient mice had lower levels of TGF‐β. This suggests that DCs secrete factors such as TGF‐β that are important for other cells during wound healing. Interestingly, another study using DC‐depleted mice in a model of myocardial infarction showed that, in the absence of DCs, the ratio of M1:M2 macrophages in the infarcted area was higher than that of control mice (Anzai et al., 2012). This means that DCs normally promote a repair phenotype in macrophages after tissue damage.

Due to their role in tissue repair, it is not surprising that DCs are also associated with pulmonary fibrosis. It was reported that large numbers of cDCs accumulate within fibroblast foci in lungs of patients with pulmonary fibrosis (Marchal‐Sommé et al., 2007). A mouse model of bleomycin‐induced pulmonary fibrosis showed that during the inflammatory phase monocytes recruited into the lung rapidly differentiated into DCs. Subsequently, higher numbers of mature DCs expressing MHCII, CD40, CD83, and CD86 surface molecules were found in lung tissue during the repair phase compared to the inflammatory phase (Bantsimba‐Malanda et al., 2010). This suggests that DCs, just as described before, are important in the repair phase possibly because of their effect on other immune cells. Moreover, DCs are also thought to play a role in fibrosis through communication with fibroblasts. Recently, DCs and fibroblasts were found to co‐localize within the renal interstitium in human kidney, suggesting the possibility of direct crosstalk between the two cell types (Dixon, Rossmann, Kamerling, & van Kooten, 2014). In addition, lung fibroblasts were shown to have a crucial role in regulating both fibrotic and immune responses by secreting chemokines that direct DC trafficking from the lung to the mediastinal lymph node through TGF‐β αvβ8‐mediated activation (Kitamura et al., 2011). Freynet and colleagues demonstrated that co‐culture of human lung fibroblasts (from both control and IPF lung tissue) with immature DCs resulted in lower expressions of MHCII, CD86, and CD83 on DCs with a concomitant decrease in their T cell stimulatory activity (Freynet et al., 2016). This suggests that lung fibroblasts act as immunoregulatory cells able to modulate the maturation of DCs by maintaining them in an immature state within fibrotic lesions, which could inhibit T cell proliferation. This is of importance as several studies have shown that DCs play a critical role in bleomycin‐induced pulmonary fibrosis by regulating T cell activation (Bayes, Bicknell, MacGregor, & Evans, 2014; Pociask et al., 2011; Trujillo, Hartigan, & Hogaboam, 2010). DCs in bleomycin‐exposed lung tissue express high levels of CD86, which can result in activation of T cells through CD28. In fact, studies with CD28‐deficient mice indicate that T cell co‐stimulation via CD28 is crucial for the development of bleomycin‐induced pulmonary fibrosis (Okazaki et al., 2001). These studies suggest that mature DCs play a role in pulmonary fibrosis by providing co‐stimulatory signals (CD28−CD86) for T cell activation.

In conclusion, DCs are important during tissue repair and play a potential role in pulmonary fibrosis. However, how DCs contribute to fibrogenesis is still poorly understood. The evidence thus far supports the idea that DCs may contribute to a repair response in fibrosis by the interaction and subsequent influence on fibroblasts, macrophages, and T cells. These interactions still require further investigation.

#### 3.1.4 Fibrocytes

Fibrocytes are circulating bone‐marrow‐derived myeloid cell progenitors that can differentiate into fibroblasts and myofibroblasts once they enter tissues. Upon tissue damage, fibroblasts concentrate in the area of injury under the influence of chemokines and cytokines produced by the injured tissue. They contribute to rebuilding tissue by producing high quantities of ECM components (Gabbiani, 2003; Midwood, Williams, & Schwarzbauer, 2004). This process intensifies after they transform into contractile myofibroblasts expressing alpha smooth muscle actin and secreting even more ECM components, which allows for wound contraction. However, myofibroblasts are also the most important cells responsible for the excess production of ECM in fibrotic processes and, at least in the case of pulmonary fibrosis, they can originate from monocyte‐derived fibrocytes (Andersson‐Sjöland et al., 2008; Kendall & Feghali‐Bostwick, 2014).

In 1994, fibrocytes were first described as circulating monocyte‐derived cells that migrate into sites of tissue injury and express a fibroblast‐like phenotype in scar‐tissue‐like lesions such as cardiovascular disease, pulmonary fibrosis, and even normal ageing (Bucala, Spiegel, Chesney, Hogan, & Cerami, 1994). Circulating fibrocytes comprise approximately 0.1−0.5% of leukocytes in peripheral blood and they rapidly enter tissues and contribute to tissue remodeling after injury (Dupin et al., 2016; Herzog & Bucala, 2010). To mediate their entry into inflamed tissues after damage, fibrocytes express the chemokine receptors CCR2, CCR7, and CXCR4 (Dupin et al., 2016; Ekert et al., 2011; Fu et al., 2015).

Fibrocytes are a unique population of immune cells because they possess characteristics both of hematopoietic cells and of mesenchymal cells (Reese et al., 2014; Suga et al., 2014). They express the hematopoietic cell markers CD34, CD45, FcγR, LSP‐1, and MHCII, but they also express stromal cell markers such as collagens, fibronectin, and MMPs (Peng & Herzog, 2012). It is currently accepted that the minimum number of markers necessary for identifying fibrocytes in culture, in tissue sections, or in the circulation are the expressions of collagen I and CD45 (Fujiwara et al., 2012). However, due to their apparent plasticity and the lack of consensus on other cellular features, such as granularity, there is still a need for an accurate gating approach for the further characterization of functional aspects of fibrocytes.

Previous studies reported that patients with pulmonary fibrosis have higher percentages of circulating fibrocytes than healthy individuals (6−10% vs. 0.5%) (Mehrad et al., 2007). This has been reported in other fibrotic diseases as well, and therefore it appears that there is a pivotal link between fibrocytes and wound healing and fibrosis (Grieb & Bucala, 2012; Kendall & Feghali‐Bostwick, 2014). There are several possible explanations of how these high percentages of fibrocytes could play a role in wound healing and fibrotic processes.

First, fibrocytes themselves could produce essential ECM proteins that are involved in wound repair and fibrosis. Reports show that fibrocytes are a source of lung fibroblasts in IPF and can differentiate into myofibroblasts once they enter into lung tissue (Andersson‐Sjöland et al., 2008). Fibrocytes were shown to express numerous ECM molecules, including vimentin, fibronectin, and collagens (Fan, Takawale, Lee, & Kassiri, 2012). Nonetheless, in comparison to fibroblasts, human fibrocytes have a matrix‐stabilizing function, as opposed to the predominant matrix‐building function of tissue fibroblasts (Bianchetti et al., 2012). Second, fibrocytes could drive proliferation, migration, and ECM production by resident fibroblasts. Prior studies proposed that collagen I produced by fibrocytes could promote fibroblast activation (Chesney, Metz, Stavitsky, Bacher, & Bucala, 1998). Fibrocytes may also influence resident epithelial or mesenchymal cells to differentiate into myofibroblasts by the production of important profibrotic cytokines (e.g., TNF‐α, IL‐6, IL‐10), chemokines (e.g., CCL3, CCL4, CCL2, CXCL8, and growth‐related oncogene‐alpha) and growth factors (e.g., M‐CSF, TGF‐β, platelet‐derived growth factor [PDGF], insulin‐like growth factor‐1, angiogenin) (Andersson‐Sjöland et al., 2008; Bellini & Mattoli, 2007; Grieb & Bucala, 2012; Schmidt, Sun, Stacey, Mori, & Mattoli, 2003). Third, a positive feedback loop between fibroblasts and fibrocytes may enhance fibrosis. Pilling and colleagues showed that fibroblasts stimulated with fibrocyte‐secreted TNF‐α can secrete lumican, which in turn acts directly on monocytes to differentiate into fibrocytes (Pilling, Vakil, Cox, & Gomer, 2015). In addition, the level of lumican was much higher in both human pulmonary fibrotic lesions and in a mouse model of bleomycin‐induced pulmonary fibrosis (Pilling et al., 2015). These results may explain why fibrocytes are rarely identified in healthy lung, heart, and liver tissue, but are easily detected in fibrotic lesions. Lastly, fibrocytes could be involved in the stimulation of angiogenesis via the production of MMP9, vascular endothelial growth factor (VEGF), basic fibroblast growth factor (bFGF), CXCL8, and PDGF (Garcia‐de‐Alba et al., 2010; Hartlapp et al., 2001; Smadja et al., 2014).

Pulmonary fibrosis has a high mortality rate with few effective drugs and even fewer reliable biomarkers for clinical management of patients. In 2009, several studies demonstrated that circulating fibrocytes could be a potential biomarker in pulmonary fibrosis predicting the progression of the disease (Lapar et al., 2011; Moeller et al., 2009; Strieter, Keeley, Burdick, & Mehrad, 2009). They found that the survival rate of patients with pulmonary fibrosis with more than 5% fibrocytes of total blood leukocytes was around 7 months, whereas patients with less than 5% fibrocytes lived for an average of 27 months after establishing fibrocyte percentages. Of the drugs against pulmonary fibrosis currently on the market, both pirfenidone and nintedanib were shown to affect fibrocytes which could thus be an explanation for their beneficial effects in patients with pulmonary fibrosis (Axell‐house et al., 2017). Pirfenidone was shown to diminish the fibrocyte pool and the migration of these cells in bleomycin‐induced pulmonary fibrosis in mice (Inomata et al., 2014), and for nintedanib it was found that it inhibits the migration and differentiation of fibrocytes induced by growth factors in vitro (Sato et al., 2017). The number of fibrocytes in the bleomycin‐induced pulmonary fibrosis model was reduced by the administration of nintedanib and this was associated with antifibrotic effects (Sato et al., 2017).

An inhibitor of monocyte‐to‐fibrocyte differentiation, known as PRM‐151 (recombinant human serum amyloid pentraxin 2), is currently being developed and tested as a potential treatment for pulmonary fibrosis (Dillingh et al., 2013). Pentraxin 2 was also found to promote differentiation of monocytes into regulatory macrophages, and preclinical studies using recombinant pentraxin 2 in mice and rats have shown a reduction in bleomycin‐induced pulmonary fibrosis. The first clinical applications have shown that PRM‐151 has antifibrotic effects and is well tolerated with a favorable pharmacokinetic profile without serious adverse reactions (Van Den Blink et al., 2016).

In summary, fibrocytes are connected to fibrotic processes through their differentiation into myofibroblasts, their promotion of local (myo)fibroblast differentiation, and their regulation of other processes like angiogenesis. However, the lack of a standard technique for the accurate characterization of fibrocytes hampers the identification of their biological functions in pathological conditions. More studies are necessary to determine the exact functional and phenotypical overlap between the myofibroblasts originating from fibrocytes and from fibroblasts and the contribution of each type to fibrotic diseases.

### 3.2 Granulocytes

#### 3.2.1 Neutrophils

Neutrophils are polymorphonuclear leukocytes that originate from bone marrow myeloid progenitors under the influence of granulocyte colony stimulating factor (G‐CSF) (Lieschke et al., 1994). They are considered as short‐lived cells with a lifespan of about 12.5 h in mice and 5.4 days in humans, although their longevity increases up to sevenfold once they are activated (Pillay et al., 2010; Summers et al., 2010). In steady state, neutrophils can be found in bone marrow, spleen, liver, and lung (Peters, 1998; Summers et al., 2010). The reason why neutrophils concentrate in these tissues is not completely understood and it has been suggested that these organs could function as a reservoir of mature neutrophils (Kolaczkowska & Kubes, 2013). Interestingly, the lung seems to contain a particularly great number of mature neutrophils (Sibille & Marchandise, 1993). Based on lung intravital microscopy studies, these neutrophils appear to be crawling and patrolling the lung vasculature, although the exact purpose of this behavior remains to be elucidated (Kreisel et al., 2010).

Neutrophils are typically the first cells to arrive at an injured tissue. This is to be expected as neutrophils are the most abundant leukocytes in human blood and they normally function as a containing barrier for potential infectious threats (Kolaczkowska & Kubes, 2013; Yang, Feng, Zhang, Lu, & Zhao, 2016). The antimicrobial functions of neutrophils consist of phagocytosis of microbes, exocytosis of enzymes and antimicrobial compounds, production of reactive oxygen species, and the release of neutrophil extracellular traps (NETs) (Brinkmann et al., 2004; Segal, 2005).

In addition to their antimicrobial function, neutrophils can phagocytose tissue debris produced by injury and they can promote the healing process by recruiting more neutrophils and other leukocytes through the production of mediators such as IL‐17, leukotriene B4, VEGF‐A, and chemokines (Kolaczkowska & Kubes, 2013; Wilgus, Roy, & McDaniel, 2013; Zemans et al., 2011). It was initially believed that neutrophils, shortly after fulfilling their function in injured tissue, would die by apoptosis and would be phagocytosed by macrophages (Buckley, Gilroy, Serhan, Stockinger, & Tak, 2012). However, Hughes and colleagues showed that neutrophils do not always die by apoptosis and have an alternative fate known as “reversed migration.” This process involves the migration of neutrophils away from the injured area and ultimately re‐entering the vasculature (Hughes et al., 1997). It is still unclear whether this behavior is associated with a change in the activation status of neutrophils that could lead to a spread of the inflammation. Uncontrolled activation of neutrophils can hamper the healing process, which could lead to pathological conditions. It has been shown that altered numbers and increased activity of neutrophils can contribute to the pathogenesis of lung diseases such as asthma and COPD (Deckers, Madeira, & Hammad, 2013; Grabcanovic‐Musija et al., 2015; Haldar & Pavord, 2007; Holgate, 2012; Pedersen et al., 2015; Qiu et al., 2003; Wright et al., 2016).

Neutrophils were also shown to be important in the context of pulmonary fibrosis. In IPF, high percentages of neutrophils in bronchoalveolar lavage fluid of patients correlated to early mortality (Kinder et al., 2008). In addition to high neutrophil counts, neutrophil activity was found to be altered in pulmonary fibrosis; biopsies from patients with pulmonary fibrosis show NET expression close to myofibroblasts and neutrophil elastase levels are higher in lavage fluid and plasma of IPF patients compared to nonsmoker controls (Chrysanthopoulou et al., 2014; Obayashi et al., 1997). Interestingly, despite the ECM degrading ability of neutrophil elastase it also appears to be contributing to the pathogenesis of pulmonary fibrosis. A study with neutrophil‐elastase‐deficient mice showed that its absence was associated with an attenuation of the characteristics of pulmonary fibrosis (Chua et al., 2007). This is supported by another murine study that demonstrated that neutrophil‐elastase‐deficient mice present a significant reduction in fibroblast and myofibroblast accumulation in comparison to wild type mice and that elastase deficiency protected mice from asbestos‐induced pulmonary fibrosis (Gregory et al., 2015). Moreover, a mouse model of pulmonary fibrosis induced by chronic fungal infection with *Paracoccidioides brasiliensis* showed that depletion of neutrophils could attenuate pulmonary fibrosis and inflammation (Puerta‐Arias, Pino‐Tamayo, Arango, & Gonzalez, 2016). This suggests that, at least in these models, the pro‐inflammatory activity of neutrophils, possibly through the production of neutrophil elastase and other compounds, can promote tissue injury leading to a continuous activation of myofibroblasts in an attempt to heal the tissue, therefore promoting fibrosis.

It is clear from these and other recent studies that neutrophils may have a bigger impact on wound healing and fibrosis development than just being the first responders to tissue injury, as they may play roles beyond phagocytosis and immune cell recruitment (Yang et al., 2016). Neutrophil apoptosis alone may skew the polarization of macrophages towards an M2 phenotype with increased IL‐10 and decreased IL‐12 and IL‐23 production, thus promoting tissue repair (Filardy et al., 2010). Moreover, in the past years studies have shown that neutrophils themselves can polarize to N1/N2 profiles (pro‐inflammatory/anti‐inflammatory), similar to the polarization observed in macrophages (Fridlender et al., 2009). So far, these neutrophil phenotypes have mostly been studied in the context of tumors and their roles and implications in fibrosis are, to our knowledge, still unknown. These interesting discoveries show that neutrophils may be more important than usually considered and therefore it might be of interest for future studies to focus on the role of this cell type in the pathogenesis of fibrotic diseases.

#### 3.2.2 Eosinophils

Eosinophils are part of the granulocyte family and account for 1−3% of all leukocytes (Eltboli & Brightling, 2013). They originate from the common myeloid progenitor in bone marrow via stimulation with IL‐3, IL‐5, and GM‐CSF (Denburg, 1999; Wardlaw, Brightling, Green, Woltmann, & Pavord, 2000). This stimulation leads to their maturation to eosinophils and their migration to blood. In blood, mature eosinophils have a short lifespan of about 8−18 h, but it can be extended to 2−14 days depending on the tissues they reach and the cytokines present in that niche (Park & Bochner, 2010). Once in blood they can be recruited to the lung under the influence of CC chemokines (also known as eotaxins), such as CCL11 (Uhm, Kim, & Chung, 2012). In lung tissue, the function of eosinophils is thought to be regulated by Th2 cells via the production of IL‐4, IL‐5, and IL‐13, which makes eosinophils bind strongly to airway vessels and survive apoptosis (Saha & Brightling, 2006; Symon et al., 1996; Wardlaw, 1999; Woltmann, McNulty, Dewson, Symon, & Wardlaw, 2000).

Eosinophils have mostly been associated with immune responses against helminths and other pathological organisms and with Th2‐related inflammatory responses (Acharya & Ackerman, 2014; Uhm et al., 2012). They exert their functions through production of granules containing major basic protein, eosinophil cationic protein, eosinophil peroxidase, and eosinophil‐derived neurotoxin (Acharya & Ackerman, 2014). Although these compounds aim to kill pathogens, they are also toxic to host cells, which is probably why they contribute to airway inflammation. High numbers of eosinophils and high levels of proteins from their granules have been associated with lung diseases such as asthma and COPD (Eltboli & Brightling, 2013).

In the case of pulmonary fibrosis, a series of studies in the last three decades have connected eosinophils to pulmonary fibrosis by evaluating the cellular composition of lavage fluid and sputum of pulmonary fibrosis patients and control individuals. These reports show that eosinophil counts and activity (i.e., levels of eosinophil cationic protein) are higher in pulmonary fibrosis patients in comparison to control individuals (Birring et al., 2005; Fujimoto, Kubo, Yamaguchi, Honda, & Matsuzawa, 1995; Hällgren, Bjermer, Lundgren, & Venge, 1989; Peterson, Monick, & Hunninghake, 1987; Rudd, Haslam, & Turner‐Warwick, 1981; Watters et al., 1987). The high numbers of eosinophils in pulmonary fibrosis lungs are thought to be associated with the expression of TNF‐α and possibly also with the release of eosinophil chemotactic activity by lung fibroblasts and bronchial epithelial cells (Sato, Koyama, & Robbins, 2000; Zhang, Gharaee‐Kermani, McGarry, Remick, & Phan, 1997). Zhang and colleagues showed that, in a mouse model of bleomycin‐induced fibrosis, neutralizing TNF‐α resulted in lower eosinophil numbers and less development of pulmonary fibrosis (i.e., lower hydroxyproline content). However, it could be argued that this study provides evidence of the importance of TNF‐α in the inflammatory responses that precede fibrosis in the bleomycin model rather than evidence for the role of eosinophils in fibrosis. This is supported by the work of Hao and colleagues in which they show that fibrosis development is independent of eosinophilic infiltration (Hao, Cohen, Jennings, Bryson, & Kaplan, 2000). These authors showed that genetic deficiency or depletion of IL‐5 resulted in significantly lower eosinophil counts and activity, but development of bleomycin‐induced pulmonary fibrosis was not affected by the absence of eosinophils. This suggests that even if eosinophils migrate to the lung before the establishment of fibrosis, they do not seem to contribute to the actual fibrotic process.

The strongest evidence connecting eosinophils to pulmonary fibrosis is the fact that they are present in lavage fluid and sputum of pulmonary fibrosis patients (Birring et al., 2005; Fujimoto et al., 1995; Hällgren et al., 1989; Peterson et al., 1987; Rudd et al., 1981; Watters et al., 1987). This does not necessarily mean that they contribute significantly to fibrosis development. It is possible that they have a small contribution, as they are known for producing TGF‐β and stimulating fibroblast proliferation in vitro (Levi‐Schaffer et al., 1999). It is also possible that the cytokines produced during the inflammatory or repair phase lead to eosinophil recruitment as a bystander effect and that the correlation observed between eosinophil numbers and lung dysfunction in pulmonary fibrosis is just reflecting the chronic state of immune dysregulation in the lungs of these patients and is not a causal relation.

To summarize, eosinophils are clearly involved in some types of lung inflammation, but their role in and contribution to pulmonary fibrosis is unclear.

#### 3.2.3 Mast cells

Mast cells originate from hematopoietic stem cells in the bone marrow but complete their differentiation and maturation from mast cell progenitors in peripheral tissues (Dahlin & Hallgren, 2015). Mast cell progenitors are currently thought to be directly derived from multipotential progenitors or from common myeloid progenitors in bone marrow (Kosanovic et al., 2015). Additionally, they may originate from the granulocyte/macrophage progenitors in spleen (Kosanovic et al., 2015). They circulate in blood in their immature form only and are recruited from blood to tissue mainly through CCR2, CXCR2, CC5R, CCL2, IL‐4, and IL‐12p40. Subsequently, IgE, stem cell factor (SCF), IL‐3, IL‐4, IL‐6, IL‐9, and IL‐33 are involved in the differentiation and maturation of mast cells in tissues (Dahlin & Hallgren, 2015; Okayama & Kawakami, 2006).

Mast cells possess intracellular granules containing histamine, heparin, cytokines, proteoglycans, and proteases like tryptase and chymase among others (Overed‐Sayer, Rapley, Mustelin, & Clarke, 2014). Based on whether they express tryptase and/or chymase, they can be subdivided into two major subsets of mature mast cells in both human and rodents: mast cells containing only chymase and mast cells containing both chymase and tryptase (Dahlin & Hallgren, 2015).

In lung tissue, mast cells expressing mainly chymase are usually found in the lamina propria and adventitia of small airways and in between the bronchial epithelium and are called mucosal mast cells (Andersson, Mori, Bjermer, Lofdahl, & Erjefalt, 2010). Mast cells expressing chymase and tryptase are found in the connective tissue of smooth muscle and in the lamina propria of small airways and are therefore called connective tissue mast cells (Overed‐Sayer et al., 2014). It was reported that mucosal mast cells could transform into connective tissue mast cells when co‐cultured with human airway epithelium (Martin et al., 2012).

Mast cells express two important receptors for their activation, namely the high affinity IgE receptor (FcεR1) and the c‐kit receptor. The former leads to mast cell activation by crosslinking of antigen‐specific IgE bound to FcεR1 receptors by antigens, while the latter results in stimulation after binding of its ligand SCF (Edwards et al., 2005; Fireman, Kivity, Shahar, Reshef, & Mekori, 1999). In addition to releasing their granular content, activated mast cells also secrete a variety of cytokines (e.g., IL‐3, IL‐4, GM‐CSF, TNF‐α, TGF‐β), chemokines (e.g., CCL2, CXCL8, and CCL5), and growth factors (e.g., bFGF, VEGF, and PDGF) (Fireman et al., 1999; Krystel‐Whittemore, Dileepan, & Wood, 2016). Due to the production of histamine and the presence of the FcεR1 receptor, mast cells are key players in allergic diseases and have been associated predominantly with asthma (Galli & Tsai, 2013; Yip, Lau, & Wise, 2011).

Additionally, mast cells play a critical role in normal tissue repair through enhancing acute inflammation, promoting the proliferative phase of healing, and augmenting scar formation. After injury, activated mast cells immediately release several pro‐inflammatory mediators to recruit other immune cells, such as neutrophils, into the wound (Hart, 2001). Mast cells can also contribute to the proliferative phase by secreting several growth factors such as VEGF, bFGF, and PDGF to stimulate endothelial cells (Torres et al., 2002; Zhang et al., 2011). Furthermore, during remodeling, mast cells promote fibroblast migration, proliferation, and differentiation into myofibroblasts through production of TGF‐β, PDGF, tryptase, and histamine (Artuc, Steckelings, & Henz, 2002).

To date many studies have suggested that mast cells are involved in fibrotic processes (Aldenborg, Nilsson, Jarlshammar, Bjermer, & Enerback, 1993; Chanez et al., 1993; Walls, Bennett, Godfrey, Holgate, & Church, 1991). Three decades ago, it was already reported that there are high numbers of mast cells in lung biopsies of patients with fibrotic lung disorders (Kawanami, Ferrans, & Fulmer, 1979). Moreover, high numbers of activated mast cells were observed in close proximity to fibroblast foci in pulmonary fibrosis lung samples and it was therefore hypothesized that the interaction of mast cells and fibroblasts in pulmonary fibrosis may be crucial for fibrosis development. This interaction could be mediated by chymase as it acts as an angiotensin II‐forming enzyme that converts angiotensin I to angiotensin II. Angiotensin II has been found to stimulate fibroblast proliferation and therefore high chymase production could promote fibroblast proliferation (Urata, Kinoshita, Misono, Bumpus, & Husain, 1990). Interestingly, chymase inhibition has been shown to attenuate pulmonary fibrosis by decreasing TGF‐β expression and diminishing chymase‐induced fibroblast proliferation (Kosanovic et al., 2015; Takato et al., 2011; Tomimori et al., 2003). Furthermore, Wygrecka and colleagues found higher levels of the mast cell activator SCF in IPF lung and IPF primary fibroblasts compared to healthy control subjects (Wygrecka et al., 2013). In their study, they co‐cultured lung fibroblasts from IPF patients with mast cells and found that SCF produced by fibroblasts enhanced mast cell survival and proliferation. Additionally, activated/degranulated mast cells were shown to release high levels of tryptase, which increased lung fibroblast proliferation in a protease‐activated receptor‐2‐dependent manner (Villar et al., 2015). Together, the evidence suggests that there is a positive feedback loop between fibroblasts and mast cells in fibrotic lung tissue, which could contribute to the fibrotic process.

In summary, mast cells play an important role in hypersensitivity and allergic responses and they may also be important contributors to fibrotic disease. The interactions between mast cells and fibroblasts may be the most likely way of contributing to fibrosis development.

#### 3.2.4 Basophils

Basophils are the rarest type of granulocytes. They are estimated to account for less than 1% of peripheral blood leukocytes and they also have a short lifespan of around 2 days (Karasuyama & Yamanishi, 2014; Ohnmacht & Voehringer, 2009). Their development begins with the common myeloid progenitor in bone marrow after stimulation with IL‐3, IL‐5, SCF, thymic stromal lymphopoietin, and GM‐CSF (Arock, Schneider, Boissan, Tricottet, & Dy, 2002; Siracusa et al., 2011). Mature basophils migrate to blood from which they can be recruited to tissues by an inflammatory response (Karasuyama & Yamanishi, 2014). Little is known about which chemokines are responsible for basophil migration to tissues.

Basophils are well known for producing high levels of IL‐4, IL‐13, and histamine and for expressing high affinity IgE receptors, which is why they, like mast cells, associate with hypersensitivity and allergic responses (Geering, Stoeckle, Conus, & Simon, 2013; Karasuyama & Yamanishi, 2014). They have also been found in injured tissues during the repair phase, although their exact role in wound healing is not clear (López, Cervero, Jiménez, & Sánchez, 2014). Nonetheless, they are known for being involved in Th2 responses by producing IL‐4, which may influence the function of monocytes, macrophages, and other immune cells (Karasuyama & Yamanishi, 2014; Min et al., 2004; Voehringer, Shinkai, & Locksley, 2004). To be noted, studies with basophil‐deficient mice have shown that, despite their Th2 contribution, basophils are not necessary to establish a Th2 response. Ohnmacht et al. showed this in a basophil‐deficient mouse model, which showed a normal recruitment of Th2 cells and eosinophils upon infection with the helminth *Nippostrongylus brasiliensis* (Ohnmacht et al., 2010). However, in this model basophils were found to be essential in IgE‐mediated chronic allergic dermatitis and protection against a second infection with *N. brasiliensis*. The association of basophils with allergic responses and immunity against parasites has also been reported by other authors (reviewed by Karasuyama & Yamanishi, 2014; Schwartz, Eberle, & Voehringer, 2016) and has been observed in the lung as well, but little is known about their actual role in wound healing (Motomura et al., 2014; Ohnmacht et al., 2010; Wakahara et al., 2013). A study using a model of basophil‐dependent allergic skin responses showed that IL‐4 produced by basophils recruited classical Ly6C^hi^ monocytes and induced their differentiation towards M2 macrophages. These macrophages were beneficial in this model since the presence of M2 macrophages protected against allergic skin inflammation (Egawa et al., 2013). However, higher numbers or activity of M2 macrophages can also contribute to fibrotic processes and in that scenario basophils become potential promoters of fibrosis (Braga et al., 2015). In addition, basophils have been suggested to promote cardiac fibrosis (i.e., cardiac transplant rejection induced fibrosis) via IL‐4 production and the activation of myofibroblasts (Schiechl et al., 2016). Schiechl and colleagues depleted CD4+ T cells in this model of cardiac fibrosis as a way to discard the IL‐4 produced by lymphocytes and still observed development of fibrosis, possibly through basophil‐produced IL‐4. However, eosinophils are also known to produce this cytokine; therefore it is possible that in this model basophils alone or in combination with eosinophils were responsible for the development of fibrosis (Voehringer et al., 2004). This means that basophils may contribute to the fibrotic process, but further investigation is required.

To the best of our knowledge there is no evidence pointing at a role for basophils in the pathogenesis of either pulmonary fibrosis or more general fibrotic processes. A short report from 1987 mentions the presence of “basophilic cells” in the lavage fluid of lungs of IPF patients (Shindoh, Tanno, Ida, & Takishima, 1987). However, the “basophilic cells” referred to in this report were cells that were positive for basophilic intracellular granules, which could also apply to mast cells. Therefore it is not clear whether this study actually described basophils. In fact, the role of basophils in many functions and dysfunctions is still unknown because of their low frequency in blood and the absence of basophilic cell lines for experimentation (Karasuyama & Yamanishi, 2014). Research on basophils has also been affected by studies into their granulocyte fellow mast cells. Basophils and mast cells share morphological and functional features, but due to the scarcity of basophils in blood more expensive and time‐consuming isolation techniques are required, making mast cells easier to study (Chirumbolo, 2012; Crivellato, Nico, & Ribatti, 2011; Karasuyama & Yamanishi, 2014; Warner & Kroegel, 1994).

To conclude, it is still unclear what the level of involvement of basophils in wound repair is and there is not enough evidence pointing at an involvement in fibrosis in general or more particularly in the lung.

## 4 WOUND HEALING IN THE AGEING LUNG

Ageing is the process of living organisms changing over time due to internal and external forces that affect the state of their systems and organs. These changes lead to a gradual decline in body functions and commonly lead to diseases. In the lung, pulmonary fibrosis and COPD are two common age‐related diseases as their onset usually presents after middle age (Rojas et al., 2015). Although age is a known risk factor, it is not clear how it affects the pathogenesis of fibrotic diseases such as pulmonary fibrosis. The general hypothesis is that fibrosis develops after persistent low‐grade inflammation, possibly due to repetitive exposure to an insult, causing continuous tissue damage. The body attempts to fix the damage with deposition of excessive ECM, which actually remodels the tissue to an extent that its architecture and its function become impaired. However, most of the mouse studies used to formulate this hypothesis have been performed using young animals and it is not known how fibrosis develops in an ageing immune system. Nonetheless, analyzing evidence on how the aged immune system responds to other conditions could help us understand what is hampering tissue repair and promoting development of fibrosis in the ageing lung.

It is well known that the immune system changes with age and there is evidence that this phenomenon known as “immunosenescence” is contributing to the development of age‐related diseases, including pulmonary fibrosis (Boe et al., 2016; Chilosi, Carloni, Rossi, & Poletti, 2013; Frasca & Blomberg, 2016). One of the best‐known characteristics of an aged immune system is the low‐grade chronic pro‐inflammatory state known as “inflammageing” (Frasca & Blomberg, 2016). This state is associated with production of high levels of pro‐inflammatory cytokines (i.e., IL‐1β, IL‐6, TNF‐α) that are thought to contribute to many diseases and to mortality in the elderly population (Boe et al., 2016). Senescence is another important player in age‐related diseases and it has been postulated that premature ageing and cellular senescence are features of pulmonary fibrosis (Chilosi et al., 2013). Interestingly, it was reported that senescence‐associated changes in lung tissue begin to appear already in middle age (Calvi et al., 2011). Calvi *et al*. showed in a longitudinal study of ageing murine lungs that the development of airspace enlargement, an age‐related feature, is marked by an increase in oxidative stress, cell death, and elastase activation (Calvi et al., 2011). This profile imposes stress on the tissue which is followed by an immune response characterized by B‐cell activation, immunoglobulin deposition, and the infiltration of macrophages. This suggests that changes in the immune system and lung homeostasis might precede the development of age‐related diseases, although the cause and origin of these changes are still unknown.

With respect to innate immune cell alterations, the most relevant for the lung are changes to neutrophils, monocytes, and macrophages (Boe et al., 2016; Paxson et al., 2011). Neutrophils of older individuals may either have impaired migration or show a highly reactive response (Boe et al., 2016). Upon cigarette smoke exposure, for instance, aged mice have significantly higher lung infiltration of neutrophils, which is accompanied by increased levels of keratinocyte‐derived chemokine and CXCL2 in comparison to young mice (Moriyama et al., 2010). As mentioned earlier, neutrophilia could contribute to the development of pulmonary fibrosis.

Ageing was also found to be associated with phenotypic and functional changes in human monocytes (Merino et al., 2011; Hearps et al., 2012). Hearps and colleagues performed a cross‐sectional study with 146 healthy individuals and they found that age was associated with a higher proportion of intermediate CD14+CD16+ and nonclassical CD14+CD16++ monocytes (Hearps et al., 2012). Moreover, they found that all monocyte subsets from older individuals exhibited shorter telomeres, higher intracellular TNF‐α levels at baseline and after LPS stimulation (except for the classical subset), and impaired phagocytosis compared to young individuals. An in vitro study by Merino and colleagues evaluated the functional and phenotypical characteristics of the intermediate monocyte subset that was obtained by culturing human classical CD14++CD16− monocytes in the presence of GM‐CSF, IL‐4, and IL‐10 (Merino et al., 2011). They found that intermediate CD14+CD16+ monocytes, compared to the classical CD14++CD16− subset, have a senescent phenotype (i.e., higher counts and levels of β‐galactosidase) and a higher pro‐inflammatory activity in terms of chemokine receptor expression (i.e., CCR5, CCR7, CX3CR1) and adhesion to endothelial cells. Together these studies suggest that the proportion of the intermediate CD14+CD16+ monocyte subset is higher with older age and that this subset is characterized by a senescent and pro‐inflammatory phenotype. Therefore their accumulation in older individuals could be contributing to chronic inflammation and possibly to inflammatory diseases. Additionally, Suchy and colleagues showed that LPS stimulation of monocyte‐derived macrophages from young healthy individuals resulted in expression of both classical activation markers (i.e., nitric oxide and reactive oxygen species) and alternative activation markers (i.e., suppressor of cytokine signaling 1 and arginase‐1) (Suchy, Łabuzek, Bułdak, Szkudłapski, & Okopień, 2014). Conversely, in elderly healthy individuals only the classical activation markers were present, suggesting that monocyte‐derived macrophages of young individuals are more capable of eliciting a balanced response to inflammatory stimuli than the elderly.

Alveolar macrophages are also affected by ageing. For instance, they were shown to have decreased efferocytosis (phagocytosis of apoptotic cells), which can lead to a prolongation and spreading of inflammation (Aprahamian, Takemura, Goukassian, & Walsh, 2008; Arnardottir, Dalli, Colas, Shinohara, & Serhan, 2014). Moreover, alveolar macrophages from old mice were found to produce lower levels inflammatory cytokines (i.e., TNF‐α and IL‐6) in response to stimuli, which was also observed in splenic macrophages of old mice (Gomez, Goral, Ramirez, Kopf, & Kovacs, 2006; Hinojosa, Boyd, & Orihuela, 2009; Linton & Dorshkind, 2004; Murciano, Yáñez, O'Connor, Gozalbo, & Gil, 2008; Ren et al., 2009). It has been suggested that the high levels of pro‐inflammatory cytokines that characterize “inflammageing” are constantly activating macrophages to a point where further stimulation with PAMPs or other relevant stimuli is not achieved and proper responses to these stimuli is not possible anymore (Kovacs, Grabowski, Duffner, Plackett, & Gregory, 2002).

In addition to changes in immune cells, the overall regenerative capability has been shown to decrease with ageing. A study by Paxson and colleagues evaluated the regenerative ability of the lungs after pneumonectomy in young (3 months), middle‐aged (9 months), and old mice (24 months). They found that young mice were able to fully restore the lung volume and alveolar surface by day 21, while the middle‐aged mice showed a limited recovery in the same time and the old mice had a total lack of recovery (Paxson et al., 2011). The authors found significantly lower baseline numbers of type 2 alveolar epithelial cells (AECII) in older mice and even lower numbers after pneumonectomy, compared to the young mice. Since AECII are progenitor cells for the pneumocytes lining the alveoli, a decrease in AECII denotes a lower alveolar regenerative capacity after injury. Additionally, various growth factors are expressed at lower levels with age (i.e., VEGF, PDGF, epidermal growth factor [EGF]), which could contribute to a delay or a deficiency in re‐epithelialization and overall tissue regeneration (Stout & Suttles, 2005).

Altogether, the evidence suggests that ageing entails extensive changes in the immune system that influence inflammatory and repair responses in the elderly lung. These changes include dysregulation in numbers or functions of neutrophils, monocytes, and macrophages. Moreover, a decline in the proliferative and regenerative abilities of AECII contributes to the poor or altered healing response in aged lungs. Table 1 summarizes the most important findings reviewed in this section. It is not clear whether these immune changes are the cause of pulmonary fibrosis, but they may contribute to its pathogenesis and to the development of other age‐related diseases.

**Table 1:** Features of immunosenescence in the context of myeloid cells

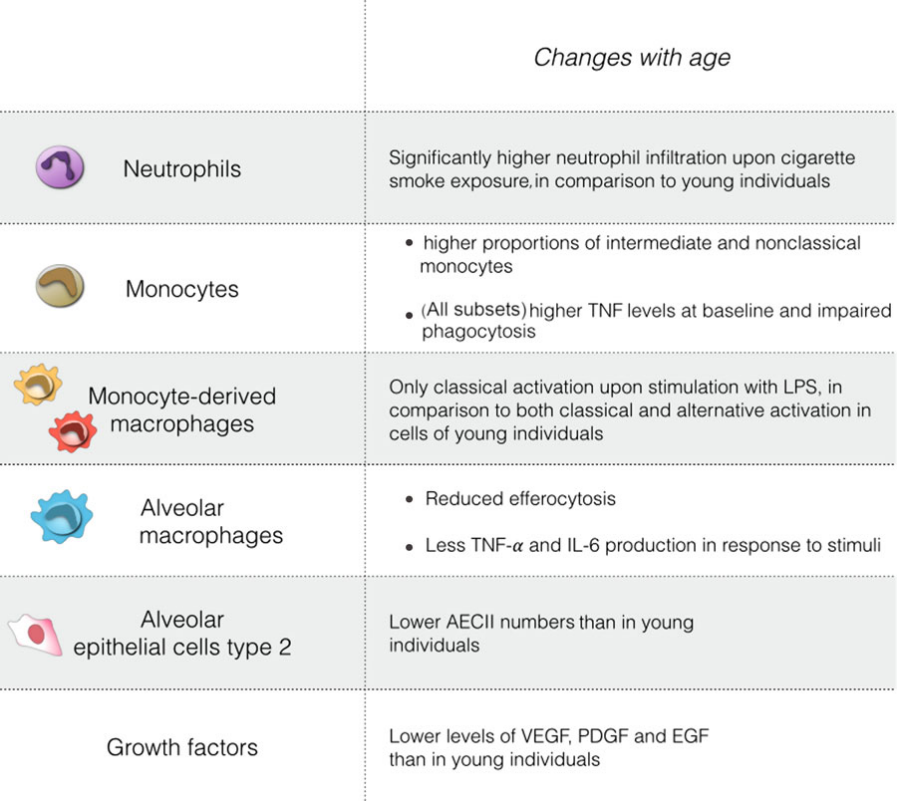

## 5 CONCLUDING REMARKS

Altogether the evidence shows that myeloid cells are not only leading players in tissue repair, but they are also tightly associated with the development of fibrosis in the lung. The evidence connecting myeloid cells with fibrosis development is strong for neutrophils, monocytes, macrophages, and fibrocytes in particular.

The dynamics of the innate immune system in the fibrotic lung suggests that fibrosis develops as an exaggerated repair phenotype following injury with continuous feedback loops among cells and a poorly balanced response leading to chronic progressive disease. Due to their common background, fibrosis and tissue repair share characteristic cellular markers, although these two processes are not equal. For instance, fibroblasts and macrophages may present a very similar phenotype during tissue repair and in fibrosis (i.e., myofibroblast activation and M2 polarization, respectively), and mediators such as TGF‐β and IL‐4 are present in both conditions. However, while in tissue repair these traits are transitory, in fibrosis they persist. Even if these phenotypes can point to the development of fibrosis, other characteristics should be taken into consideration for the accurate assessment of fibrosis development. So, when is it accurate to call it fibrosis and when is it simply a repair response? In animal models and in patients with pulmonary fibrosis it is possible to identify fibrosis through physiological and histological characteristics, such as excess ECM deposition, alterations in lung architecture, the formation of fibroblast foci, and a poor or absent pro‐inflammatory aspect. However, some of these features are more difficult to reproduce during in vitro studies, as these models cannot represent all the possible variables found in vivo. It is therefore of importance to keep this in mind when interpreting in vitro experiments, since what one could consider as a fibrotic response could simply be the activation of a repair response. The use of patient samples, human and animal precision‐cut tissue slices, and animal models can provide more accurate information about the dynamics of fibrosis development.

The inflammatory phase is not considered to play an important role in the pathogenesis of fibrosis, or at least not during advanced stages of the disease. This has been suggested by the observation that using corticosteroids for treating fibrosis can be detrimental for patients (Fujimoto, Kobayashi, & Azuma, 2015). The study from Gibbons and colleagues suggests that the undesirable effects of corticosteroid treatment could be due to a boost of M2 macrophages that are directly associated with the development of pulmonary fibrosis (Gibbons et al., 2011). In this scenario, although a pro‐inflammatory profile is inhibited by this treatment, the induction of M2 polarization may stimulate fibrosis development. We also showed this in a model for liver fibrosis, in which Kupffer‐cell‐specific delivery of the corticosteroid dexamethasone during induction of liver fibrosis exacerbated the deposition of ECM in the liver (Melgert et al., 2001). Nevertheless, the evidence included here shows that many cells and mediators with pro‐inflammatory activity positively correlate with fibrosis development. This pro‐inflammatory activity may actually be important for resolution of an injury and be a desperate attempt of the system to resolve and its failure to do so, thus correlating with the severity of the fibrotic process. In this case, the worsening or lack of improvement in patients with pulmonary fibrosis upon anti‐inflammatory treatments could be due to a decrease in the attempt to resolve the fibrosis. It still remains to be elucidated whether particular polarization stages of pro‐inflammatory cells, such as N1 or N2 neutrophils, are predominantly present in fibrosis.

The evidence supporting the essential role of innate immune cells in the development of fibrosis is currently leading to therapeutic developments targeting myeloid cells. For instance, an anti‐galectin‐3 drug called TD139 is going to enter a phase IIB trial (Hirani, Nicol, & MacKinnon, 2017). Galectin‐3 is a β‐galactoside‐binding lectin crucial for macrophage M2 polarization and was found to be expressed more in alveolar macrophages of IPF patients compared to those of control subjects (Mackinnon et al., 2008; Nishi et al., 2007). Prevention of M2 polarization through inhibiting galectin‐3 may inhibit fibrosis development as these macrophages are strongly associated with fibrosis development (Mackinnon et al., 2008).

The study of smaller animals like salamanders may also help with finding alternative therapeutic strategies against the development of fibrosis, as these amphibians have the ability of scar‐free repair with full tissue regeneration after tissue damage. Interestingly, the study by Godwin and colleagues shows that, although many features of inflammation of the salamander resemble those of mammals, these amphibians present a more dynamic response to injury with a simultaneous induction of the inflammatory and the anti‐inflammatory phases in the first 24 h (Godwin et al., 2013). Identification of similarities and differences between the amphibian and mammalian response to tissue injury may provide targets for fibrosis regulation. It is already known that one of the differences between salamanders and mammals is the fact that salamanders lack adaptive immunity. It has been suggested that this contributes to fibrosis development but it is still unclear how. It is possible that the cells of the adaptive immunity are involved in an autoimmune‐like response against lung antigens, triggered by repetitive tissue damage. In fact, features of autoimmunity have been described for IPF and are thought to contribute to IPF exacerbation, since autoantibody‐reductive therapies have been shown to improve the lung function of IPF patients during acute exacerbation (Donahoe et al., 2015). These and other studies have shown that there are indeed alterations in the adaptive immune system of pulmonary fibrosis patients compared to healthy individuals (Duncan, 2014). Nonetheless, the evidence included here also shows that innate immunity (i.e., myeloid cells) is key in the development of fibrosis. Therefore, it is of interest for future studies to elucidate the interaction between the innate and the adaptive immune system and to find the dysfunctional connection between these two parts that leads to this pathological condition in humans.

## CONFLICT OF INTEREST

The authors have no conflict of interest to declare.
